# Conditions Inducing Excessive *O*-GlcNAcylation Inhibit BMP2-Induced Osteogenic Differentiation of C2C12 Cells

**DOI:** 10.3390/ijms19010202

**Published:** 2018-01-09

**Authors:** Hanna Gu, Mina Song, Kanitsak Boonanantanasarn, Kyunghwa Baek, Kyung Mi Woo, Hyun-Mo Ryoo, Jeong-Hwa Baek

**Affiliations:** 1 Department of Molecular Genetics, School of Dentistry and Dental Research Institute, Seoul National University, Seoul 08826, Korea; hnbn9@snu.ac.kr (H.G.); 0508mina@gmail.com (M.S.); kb57@buffalo.edu (K.B.); kmwoo@snu.ac.kr (K.M.W.); hmryoo@snu.ac.kr (H.-M.R.); 2 Department of Pharmacology, College of Dentistry and Research Institute of Oral Science, Gangneung-Wonju National University, Chuncheon 25457, Gangwon-do, Korea; daedanhae@gmail.com

**Keywords:** excessive *O*-GlcNAcylation, osteogenic differentiation, Runx2, hyperglycemia

## Abstract

Hyperglycemic conditions in diabetic patients can affect various cellular functions, including the modulation of osteogenic differentiation. However, the molecular mechanisms by which hyperglycemia affects osteogenic differentiation are yet to be clarified. This study aimed to investigate whether the aberrant increase in protein *O*-linked-β-*N*-acetylglucosamine glycosylation (*O*-GlcNAcylation) contributes to the suppression of osteogenic differentiation due to hyperglycemia. To induce osteogenic differentiation, C2C12 cells were cultured in the presence of recombinant human bone morphogenetic protein 2 (BMP2). Excessive protein *O*-GlcNAcylation was induced by treating C2C12 cells with high glucose, glucosamine, or *N*-acetylglucosamine concentrations or by *O*-GlcNAc transferase (OGT) overexpression. The effect of *O*-GlcNAcylation on osteoblast differentiation was then confirmed by examining the expression levels of osteogenic marker gene mRNAs, activity of alkaline phosphatase, and transcriptional activity of Runx2, a critical transcription factor for osteoblast differentiation and bone formation. Cell treatment with high glucose, glucosamine or *N*-acetylglucosamine increased *O*-GlcNAcylation of Runx2 and the total levels of *O*-GlcNAcylated proteins, which led to a decrease in the transcriptional activity of Runx2, expression levels of osteogenic marker genes (Runx2, osterix, alkaline phosphatase, and type I collagen), and activity of alkaline phosphatase. These inhibitory effects were rescued by lowering protein *O*-GlcNAcylation levels by adding STO45849, an OGT inhibitor, or by overexpressing β-*N*-acetylglucosaminidase. Our findings suggest that excessive protein *O*-GlcNAcylation contributes to high glucose-suppressed osteogenic differentiation.

## 1. Introduction

Diabetes mellitus (DM) is a chronic metabolic disease characterized by hyperglycemia due to the inability of insulin-dependent cells to effectively take up glucose. DM frequently leads to serious complications that affect the heart, blood vessels, eyes, kidneys, and nerves. DM also significantly affects bone health. Reportedly, DM-associated hyperglycemia modulates osteoblast gene expression, function, and bone formation, thereby causing diabetic bone loss in a mouse model of insulin-dependent DM [1]. Further, enhanced formation of advanced glycation end products in the bone matrix contributes to reduced bone strength and increased fracture risk in type 2 DM [2]. Although hyperglycemic conditions in diabetic patients significantly affect bone health, the molecular mechanisms underlying the inhibition of osteogenic differentiation and bone formation remain unclear.

*O*-linked-β-*N*-acetylglucosamine (*O*-GlcNAc) glycosylation (*O*-GlcNAcylation), which involves the covalent attachment of *N*-acetylglucosamine to serine or threonine residues of proteins, is a unique post-translational modification [3,4]. More than 4000 nuclear and cytoplasmic proteins belonging to almost every functional class of proteins, including transcription factors, cytoskeletal proteins, RNA polymerases, cell cycle regulators, phosphatases, and kinases, are modified by *O*-GlcNAc [5]. The dynamics of *O*-GlcNAcylation are unique among sugar modifications, being cycled on a shorter time scale than protein turnover [6]. *O*-GlcNAcylation is highly dynamic in response to intracellular and extracellular signals, nutrient availability, and stresses, and aberrant *O*-GlcNAcylation has been implicated in the progression of diseases such as cancer, neurodegeneration, and DM [7]. Therefore, it has been suggested that global cellular *O*-GlcNAcylation levels must remain within an optimal zone in the various fluctuations of cellular environments in order to preserve normal cellular function [8].

Protein *O*-GlcNAcylation is reversibly catalyzed by *O*-GlcNAc transferase (OGT) and β-*N*-acetylglucosaminidase (OGA). OGT deletion results in embryonic lethality in mice, reflecting the significance of protein *O*-GlcNAcylation in development and survival [9]. In addition, OGA deletion is perinatally lethal in mice [10]. Reportedly, the OGA gene *MGEA5* is a DM-susceptibility locus in humans [11,12], and Goto-Kakizaki rats harboring an exon 8 deletion in *Mgea5* spontaneously develop DM [13], suggesting that aberrant upregulation of *O*-GlcNAcylation is involved in the pathogenesis and/or complications of DM. Protein *O*-GlcNAcylation increases in diabetic tissues and hyperglycemia drives excessive chronic *O*-GlcNAcylation of proteins, including those involved in insulin signaling in DM [14,15].

Reportedly, overall cellular *O*-GlcNAcylation levels change during adipogenic, chondrogenic, and osteogenic differentiation of mesenchymal stem cells (reviewed in Reference [16]). Previous reports have demonstrated that global *O*-GlcNAcylation levels increase during osteoblastic differentiation of MC3T3-E1 and bone marrow mesenchymal stem cells, and that further increases in *O*-GlcNAcylation levels by OGA inhibition promotes osteoblast differentiation [17,18,19]. Increased *O*-GlcNAcylation of Runx2, a critical transcription factor for osteoblast differentiation and bone formation, enhances transcriptional activity of Runx2 and mRNA expression levels of Runx2 target genes, including alkaline phosphatase (ALP) and osteocalcin, contributing to osteoblast differentiation [17,18]. 

However, considering that hyperglycemia increases protein *O*-GlcNAcylation and that aberrant upregulation of *O*-GlcNAcylation is involved in the pathogenesis of human diseases such as DM, it cannot be ruled out that excessive, aberrant *O*-GlcNAcylation of proteins induced by hyperglycemia may contribute to attenuated osteoblast differentiation and bone formation phenotypes observed in diabetic animal models or diabetic patients. Therefore, we hypothesized that although protein *O*-GlcNAcylation is necessary for the progression of osteogenic differentiation, excessive *O*-GlcNAcylation of proteins, including Runx2, would negatively regulate osteogenic differentiation. To test the hypothesis, we examined the effect of excessive *O*-GlcNAcylation inducers, including high concentrations of glucose (high glucose), glucosamine, and *N*-acetylglucosamine, or OGT overexpression on the osteogenic differentiation of C2C12 cells induced by treatment with bone morphogenetic protein 2 (BMP2). In the present study, we demonstrated that excessive protein *O*-GlcNAcylation-inducing conditions inhibit BMP2-induced osteogenic differentiation of C2C12 cells and transcriptional activity of Runx2.

## 2. Results

### 2.1. Excessive O-GlcNAcylation-Inducing Conditions Suppressed BMP2-Induced ALP Activity and Osteogenic Marker Gene Expression

**Figure 1:**
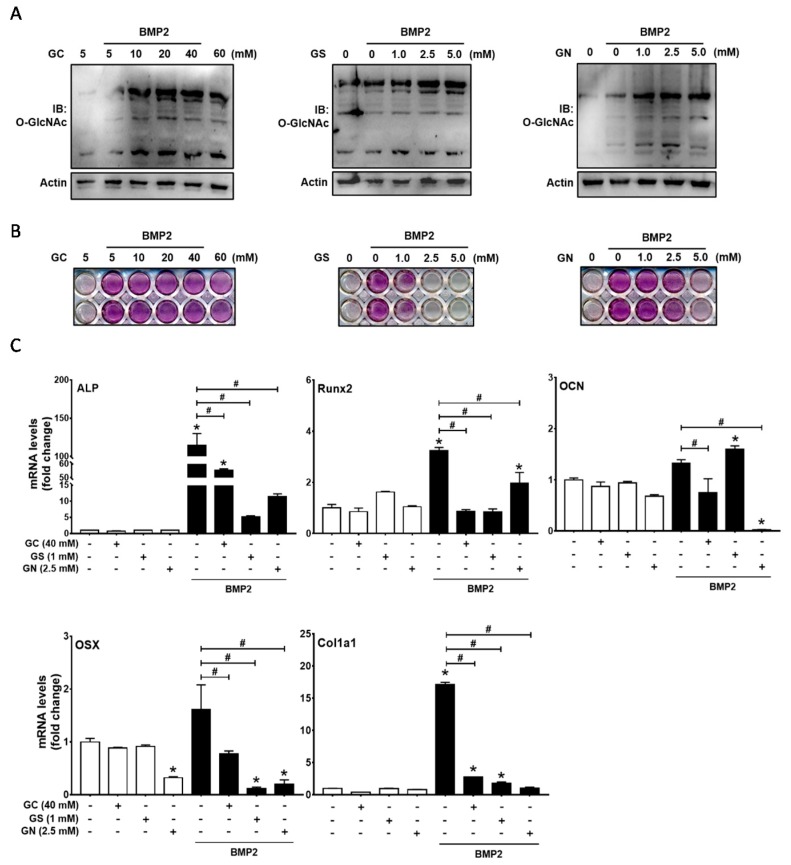
High glucose (GC), glucosamine (GS), and *N*-acetylglucosamine (GN) concentrations inhibited BMP2-induced osteogenic differentiation in C2C12 cells. (**A**) C2C12 cells were incubated for 6 h in the presence or absence of rhBMP2 (50 ng/mL) and GC, GS, or GN at the indicated concentrations, and Western blot analysis was performed to examine the levels of global protein *O*-GlcNAcylation; (**B**,**C**) C2C12 cells were incubated in the presence of the indicated reagents for 48 h, and osteogenic differentiation was examined by alkaline phosphatase (ALP) staining (**B**) and quantitative RT-PCR of osteogenic marker genes (**C**). Quantitative data are presented as mean ± SD of triplicates. * *p* < 0.05 compared with the non-treatment control; # *p* < 0.05 for the indicated pairs. OSX, osterix; OCN, osteocalcin.

C2C12 is a mouse myoblast cell line that can differentiate into osteoblasts in the presence of BMP2, thereby resulting in the induction of ALP activity and osteocalcin production [20]. To examine the effect of protein *O*-GlcNAcylation inducers on osteoblast differentiation, C2C12 cells were incubated in the presence of 50-ng/mL BMP2 and *O*-GlcNAcylation inducers. With the initiation of osteogenic differentiation, C2C12 cells were exposed to varying glucose (GC, 10, 20, 40 or 60 mM), glucosamine (GS, 1, 2.5 or 5 mM), or N-acetylglucosamine (GN, 1, 2.5 or 5 mM) concentrations for 6 h. Protein *O*-GlcNAcylation was assessed using immunoblot analysis with *O*-GlcNAc antibody. Similar to previous reports demonstrating that osteogenic stimuli increase global *O*-GlcNAcylation levels [17,18,19], BMP2 treatment moderately increased *O*-GlcNAcylation levels in C2C12 cells (Figure 1A). All three *O*-GlcNAcylation inducers further increased overall protein *O*-GlcNAcylation in C2C12 cells (Figure 1A).

Next, the progression of osteoblast differentiation was assessed after 48 h of incubation under osteogenic conditions. ALP staining demonstrated that BMP2-induced ALP activity was suppressed by glucose at concentrations of 40 and 60 mM (Figure 1B). Furthermore, glucosamine and *N*-acetylglucosamine strongly suppressed BMP2-induced ALP activity in a dose-dependent manner, and ALP staining was very weak in the presence of 2.5–5 mM glucosamine or 5 mM *N*-acetylglucosamine (Figure 1B). Therefore, subsequent experiments were performed using 40 mM glucose, 1 mM glucosamine, and 2.5 mM *N*-acetylglucosamine. To further confirm the effects on osteoblast differentiation, qRT-PCR of osteoblast marker genes was performed: ALP, type I collagen, and osteocalcin, early and late osteoblast markers, and Runx2 and osterix, critical transcription factors for osteoblastic differentiation. The expression levels of these genes were increased by BMP2 but were significantly suppressed by *O*-GlcNAcylation inducers (Figure 1C). These results suggest that conditions inducing abnormally high levels of protein *O*-GlcNAcylation inhibit BMP2-induced osteogenic differentiation of C2C12 cells.

### 2.2. Inhibition of OGT Activity Rescued BMP2-Induced Osteogenic Differentiation that was Downregulated by O-GlcNAcylation Inducers

**Figure 2:**
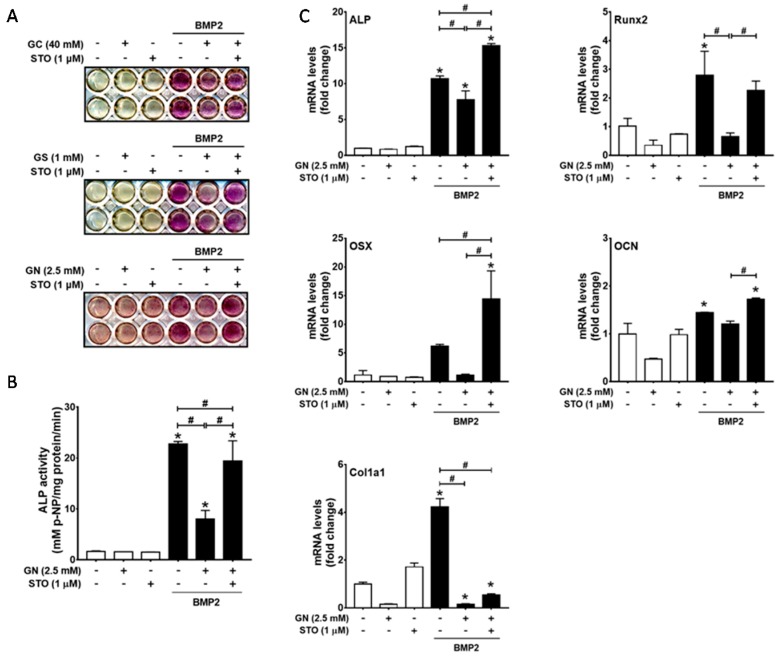
OGT activity inhibition by STO45849 (STO), an OGT inhibitor, rescued BMP2-induced ALP activity and osteogenic marker gene expression under excessive *O*-GlcNAcylation-inducing conditions. C2C12 cells were incubated for 48 h in the presence or absence of the indicated reagents, and osteogenic differentiation was examined by ALP staining (**A**); ALP activity assays (**B**); and qRT-PCR analysis of osteoblast differentiation marker genes (**C**). * *p* < 0.05 compared with the non-treatment control; # *p* < 0.05 for the indicated pairs.

To confirm whether the inhibitory effect of *O*-GlcNAcylation inducers on osteogenic differentiation depends on excessive protein *O*-GlcNAcylation, we investigated whether an OGT inhibitor rescues BMP2-induced osteogenic differentiation in the presence of *O*-GlcNAcylation inducers. The addition of STO45849 (1 μM), a specific OGT inhibitor, rescued BMP2-induced ALP activity downregulated by *O*-GlcNAcylation inducers (Figure 2A,B). Furthermore, qRT-PCR results also demonstrated that STO45849 rescued BMP2-induced mRNA expression levels of ALP, Runx2, and osterix, which were decreased by *N*-acetylglucosamine (Figure 2C). These results indicated that OGT activity is required for the inhibitory effect of *O*-GlcNAcylation inducers on osteogenic differentiation.

### 2.3. OGT Overexpression Inhibited BMP2-Induced Osteogenic Differentiation, whereas OGA Overexpression Rescued N-Acetylglucosamine-Inhibited Osteogenic Differentiation

**Figure 3:**
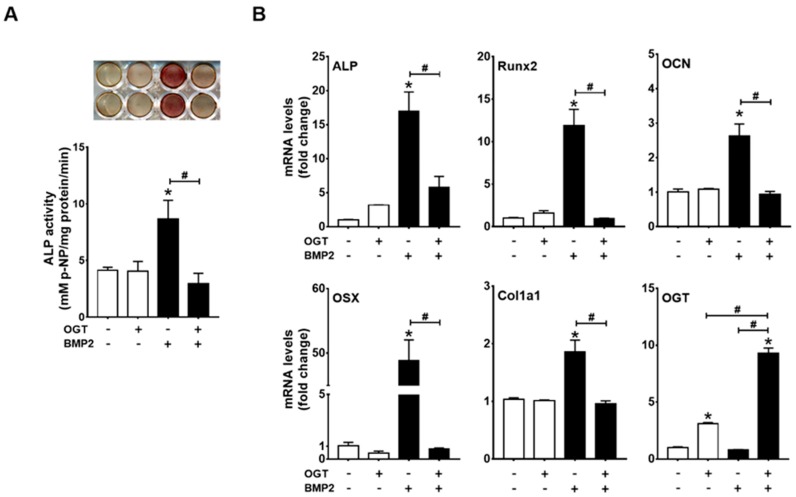
OGT overexpression inhibited osteogenic differentiation of C2C12 cells. C2C12 cells were transfected with OGT expression plasmids and incubated in the presence or absence of BMP2 for 48 h. Subsequently, ALP staining and ALP activity assays (**A**) as well as qRT-PCR analysis (**B**) were performed to examine osteogenic differentiation. * *p* < 0.05 compared with the non-treatment control; # *p* < 0.05 for the indicated pairs.

To further verify whether excessive *O*-GlcNAcylation inhibits osteogenic differentiation, we next examined the effect of OGT overexpression on BMP2-induced ALP activity and osteogenic marker gene expression. OGT overexpression blocked BMP2-induced ALP activity and osteogenic gene expression (Figure 3A,B). OGT overexpression was confirmed by RT-PCR (Figure 3B). BMP2 did not change basal OGT mRNA levels, but significantly increased OGT mRNA expression under conditions of OGT overexpression (Figure 3B).

**Figure 4:**
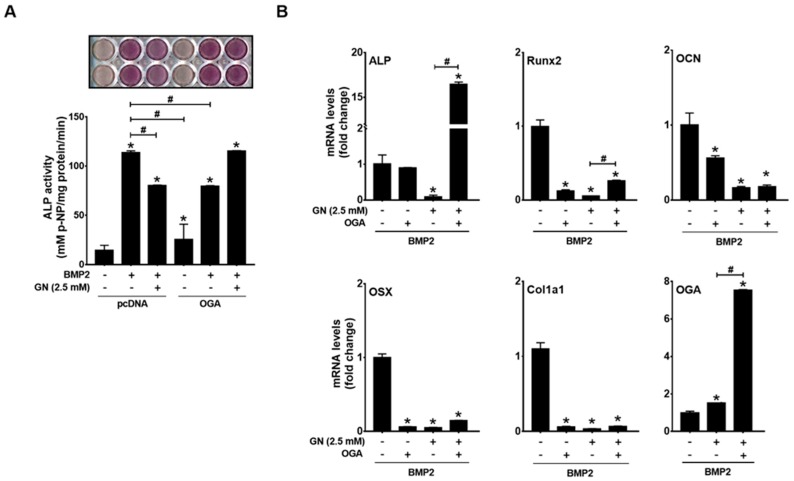
OGA overexpression rescued BMP2-induced osteogenic differentiation under excessive *O*-GlcNAcylation conditions. C2C12 cells were transfected with OGA expression plasmids and osteogenic differentiation was induced by BMP2 in the presence or absence of *N*-acetylglucosamine (GN). Subsequently, ALP staining and ALP activity assays (**A**) or qRT-PCR analysis (**B**) were performed. * *p* < 0.05 compared with the non-treatment control; # *p* < 0.05 for the indicated pairs.

We next examined whether OGA overexpression can rescue osteogenic differentiation under conditions of excessive *O*-GlcNAcylation. OGA overexpression significantly suppressed BMP2-induced ALP activity (Figure 4A) but completely rescued BMP2-induced ALP activity and ALP mRNA expression in the presence of *N*-acetylglucosamine (Figure 4A,B). *N*-acetylglucosamine-inhibited expression of Runx2, and osterix mRNA was only partially rescued by OGA overexpression (Figure 4B). OGA overexpression was confirmed by RT-PCR (Figure 4B). *N*-acetylglucosamine also increased OGA mRNA expression, which was further increased by OGA transfection (Figure 4B). These results indicated that the inhibitory effect of *N*-acetylglucosamine on BMP2-induced expression of osteoblast marker genes depends on protein *O*-GlcNAcylation.

### 2.4. Excessive O-GlcNAcylation Suppressed Runx2 Transcriptional Activity, Which Was Restored by OGT Inhibitor Addition or OGA Overexpression

**Figure 5:**
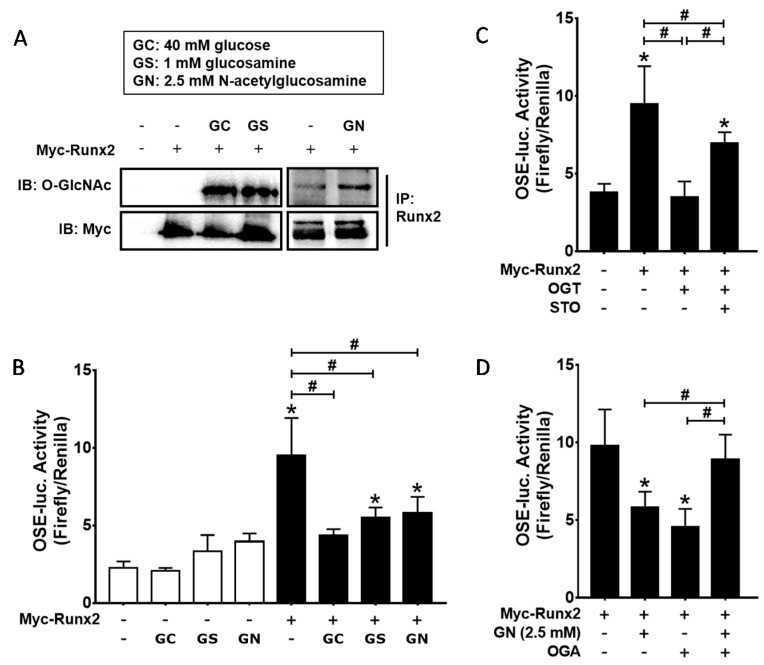
Excessive *O*-GlcNAcylation inducers suppressed Runx2 transcriptional activity, which was restored by OGT inhibitor (STO) addition or OGA overexpression. (**A**) C2C12 cells were transiently transfected with Myc-Runx2 expression plasmids and incubated under the indicated conditions for 24 h. Subsequently, immunoprecipitation with Runx2 antibody was performed, followed by immunoblotting with *O*-GlcNAc or Myc antibodies. High glucose (GC), glucosamine (GS), and *N*-acetylglucosamine (GN) enhanced Runx2 *O*-GlcNAcylation; (**B**–**D**) C2C12 cells were transiently transfected with the indicated expression plasmids and OSE-luc (a Runx2 reporter plasmid) and incubated for 24 h in the presence or absence of *O*-GlcNAcylation inducers and STO. Luciferase activity was then measured. * *p* < 0.05 compared with the non-treatment control; # *p* < 0.05 for the indicated pairs.

Although previous reports have demonstrated that Runx2 *O*-GlcNAcylation enhanced its transcriptional activity [17,18], the above results showed that excessive *O*-GlcNAcylation-inducing conditions inhibited BMP2-induced osteogenic differentiation. Therefore, we further examined whether *O*-GlcNAcylation inducers regulate Runx2 transcriptional activity in our system. To confirm Runx2 *O*-GlcNAcylation, Myc-tagged Runx2 was expressed in C2C12 cells and incubated in the presence of high glucose, glucosamine, or *N*-acetylglucosamine for 24 h. The results of the immunoblot analysis showed that Runx2 *O*-GlcNAcylation was induced by all three *O*-GlcNAcylation inducers (Figure 5A). Next, luciferase reporter assays were performed to examine whether increased Runx2 *O*-GlcNAcylation leads to changes in Runx2 transcriptional activity. C2C12 cells were transfected with the OSE-luc reporter and Myc-Runx2 expression plasmids and incubated for 24 h in the presence of 40 mM glucose, 1 mM glucosamine, or 2.5 mM *N*-acetylglucosamine. The results demonstrated that all three *O*-GlcNAcylation inducers significantly decreased Runx2 transcriptional activity (Figure 5B). Similarly, OGT overexpression blocked Myc-Runx2-mediated induction of luciferase activity, and the effect of OGT overexpression was attenuated by adding STO45849 (Figure 5C). Furthermore, OGA overexpression rescued Runx2 transcriptional activity, which was downregulated by *N*-acetylglucosamine (Figure 5D). However, OGA overexpression itself suppressed Runx2 transcriptional activity in the absence of *N*-acetylglucosamine (Figure 5D). These results indicated that excessive *O*-GlcNAcylation inducers inhibit Runx2 transcriptional activity, but that basal *O*-GlcNAcylation levels are necessary for Runx2 transcriptional activity.

### 2.5. Excessive O-GlcNAcylation Attenuated Osteogenic Differentiation and Matrix Mineralization of Human Periodontal Ligament Cells

**Figure 6:**
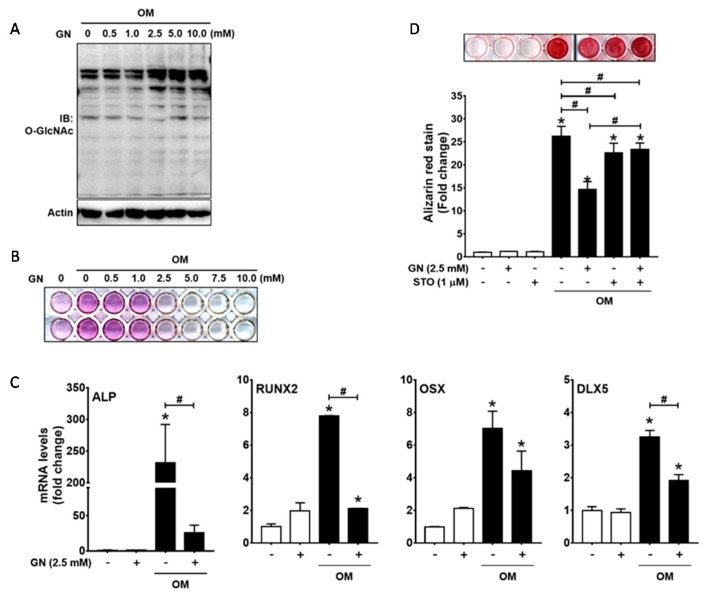
*N*-acetylglucosamine inhibits osteogenic differentiation and matrix mineralization in human periodontal ligament (hPDL) cells. (**A**) hPDL cells were incubated for 6 h in the presence of *N*-acetylglucosamine (GN) at the indicated concentrations, and Western blot analysis was performed to examine the levels of protein *O*-GlcNAcylation; (**B**,**C**) Cells were incubated for seven days and osteogenic differentiation was examined by ALP staining (**B**) and qRT-PCR of osteogenic marker genes (**C**); (**D**) hPDL cells were incubated for 21 days in the presence or absence of the indicated reagents, and Alizarin red S staining and quantification were performed. * *p* < 0.05 compared with the non-treatment control; # *p* < 0.05 for the indicated pairs. OM, osteogenic medium.

To ensure that excessive *O*-GlcNAcylation inducers inhibit osteogenic differentiation, we next examined the effect of *N*-acetylglucosamine on osteogenic differentiation of primary cultured human periodontal ligament (hPDL) cells. With the initiation of osteogenic differentiation, hPDL cells were exposed to varying concentrations of *N*-acetylglucosamine (GN, 0.5, 1, 2.5, 5, or 10 mM) for 6 h. Increased global protein *O*-GlcNAcylation levels was induced by treatment with *N*-acetylglucosamine at the concentrations of 2.5, 5, and 10 mM (Figure 6A). Next, the progression of osteoblast differentiation was assessed after seven days of incubation under osteogenic conditions. ALP staining demonstrated that ALP activity was suppressed by *N*-acetylglucosamine at concentrations of 2.5–10 mM (Figure 6B). qRT-PCR of early osteoblast marker genes demonstrated that the expression levels of ALP, RUNX2, and DLX5 genes were increased by osteogenic stimuli, but were significantly suppressed by *N*-acetylglucosamine (Figure 6C). To further examine the effect of *N*-acetylglucosamine on matrix mineralization, hPDL cells were incubated for 21 days, followed by Alizarin red S staining (Figure 6D). *N*-acetylglucosamine significantly suppressed osteogenic stimuli-induced matrix mineralization, which was rescued by the addition of STO45849. However, STO45849 treatment alone inhibited matrix mineralization. These results suggest that conditions inducing abnormally high levels of protein *O*-GlcNAcylation inhibit osteogenic differentiation of hPDL cells.

## 3. Discussion

DM is a common metabolic disorder characterized by hyperglycemia, insulin resistance, and relative insulin deficiency [21]. DM is a complex syndrome with more than one cause and is responsible for numerous complications that affect the entire body. Increasing evidence has demonstrated that DM can cause bone metabolism disorder, leading to decreased bone mineral density and defective bone healing [22,23,24]. Under hyperglycemic conditions, the unbalanced coupling of bone resorption and formation in remodeling processes promotes bone loss [25].

Previously, we observed that hyperglycemia increases sclerostin expression by enhancing reactive oxygen species and tumor necrosis factor alpha production in osteoblasts and osteocytes [26], suggesting that increased sclerostin expression contributes to DM-associated osteopenia and accelerated alveolar bone loss in diabetic patients with periodontitis. It has also been demonstrated that high glucose concentrations inhibit osteogenic differentiation of bone marrow mesenchymal stem cells by attenuating the effect of BMP2 [27]. Hyperglycemia is considered a potential contributor to diabetic osteoporosis [24], but the mechanism underlying hyperglycemia-induced bone loss is still unclear.

Previous reports have demonstrated that global protein *O*-GlcNAcylation increases during the early stages of osteoblast differentiation and correlates with increased Runx2 transcriptional activity and increased osteogenic marker gene expression levels [17,18,19]. Similarly, we also observed that BMP2 treatment moderately increased overall *O*-GlcNAcylated protein levels, which was clear even after incubation with BMP2 for 30 min. In addition, the inhibition of BMP2-induced protein *O*-GlcNAcylation by OGA overexpression significantly suppressed BMP2-induced ALP activity and Runx2 transcriptional activity. These results further support the notion that a moderate increase in protein *O*-GlcNAcylation is required for the progression of osteogenic differentiation.

However, when we further increased *O*-GlcNAcylation in the presence of BMP2 by treating cells with high glucose, glucosamine, or *N*-acetylglucosamine or by OGT overexpression, BMP2-induced ALP activity and osteoblast differentiation marker gene expression were clearly suppressed. Furthermore, *N*-acetylglucosamine treatment attenuated the expression levels of osteogenic differentiation marker genes and matrix mineralization of hPDL cells. These results are consistent with the decreased bone formation phenotype observed in type 2 DM patients. They are, however, in contrast to the results of previous studies, which showed that the upregulation of *O*-GlcNAcylation by the addition of OGA inhibitors, such as PUGNAc or thiamet G, further increases osteogenic stimuli-induced Runx2 transcriptional activity and matrix mineralization [17,19]. The reason for this discrepancy is still unclear. Considering the suggestion that global *O*-GlcNAcylation levels should be maintained within an optimal zone to preserve normal cellular function [8], excessive global *O*-GlcNAcylation may disrupt the progression of normal osteogenic differentiation in C2C12 and hPDL cells.

Runx2 is a transcription factor critical for the progression of osteoblast differentiation. Runx2 is post-translationally modified downstream of a diverse set of signaling pathways, whose coordinated action controls osteoblast differentiation and bone development [28]. Similar to previous studies [17,18], we also confirmed that Runx2 is *O*-GlcNAcylated and that its *O*-GlcNAcylation levels are increased by *O*-GlcNAcylation inducers. However, in contrast to the findings of previous studies, Runx2 transcriptional activity in our study was significantly suppressed by *O*-GlcNAcylation inducers. Reportedly, NF-κB *O*-GlcNAcylation exerts stimulatory or inhibitory effects on target gene transcription, depending on the cellular context and/or the type of *O*-GlcNAcylation-inducing conditions [29,30,31]. One possible explanation for these discrepancies is that the effects of metabolic treatments (for example, the addition of high glucose, glucosamine, or *N*-acetylglucosamine) and pharmacological treatments (for example, OGA inhibitors) are not the same: OGA inhibitors increase *O*-GlcNAcylation levels by breaking the dynamic on/off cycle, whereas metabolic treatments or OGT overexpression elevates *O*-GlcNAcylation levels by shifting the equilibrium toward modification [15]. Furthermore, other studies have demonstrated that the concentrations of UDP-GlcNAc, the donor for O-GlcNAcylation, may affect OGT substrate selectivity [32,33], suggesting that the increase in UDP-GlcNAc concentration induced by metabolic treatments may differentially regulate modification sites in Runx2 or the type of modified proteins. Furthermore, it is unclear whether the inhibitory effect of *O*-GlcNAcylation inducers on Runx2 transcriptional activity results directly from Runx2 *O*-GlcNAcylation. These are important issues that should be clarified in future studies.

In summary, we demonstrated that during BMP2-induced osteogenic differentiation, exposing C2C12 cells to excessive *O*-GlcNAcylation-inducing conditions, including high glucose, glucosamine, *N*-acetylglucosamine or OGT overexpression, led to an excessive increase in protein *O*-GlcNAcylation, which subsequently attenuated BMP2-induced osteogenic differentiation. Similarly, *N*-acetylglucosamine attenuated ALP activity and matrix mineralization in hPDL cells. These results suggest that investigating the role of excessive protein *O*-GlcNAcylation in bone cells under hyperglycemic conditions is necessary to explain pathological bone loss in diabetic patients in future studies. In this context, the consequences of in vivo chronic hyperglycemia on protein *O*-GlcNAcylation in osteoblasts and the role of excessive *O*-GlcNAcylation in the development of diabetic bone phenotype should also be evaluated in the near future using osteoblast-specific knockout of OGT and OGA in a mouse model. 

## 4. Materials and Methods

### 4.1. Cell Cultures and Reagents

C2C12 cells were maintained in Dulbecco’s modified Eagle’s medium (DMEM) supplemented with 10% heat-inactivated fetal bovine serum (FBS) and antibiotics (100-U/mL penicillin and 100-μg/mL streptomycin). To induce osteogenic differentiation, C2C12 cells were incubated in DMEM supplemented with 5% FBS and BMP2 (50 ng/mL) for 48 h. DMEM, FBS, antibiotics, and other reagents for cell culture were purchased from HyClone Laboratories (GE Healthcare Life Sciences, South Logan, UT, USA). Recombinant human BMP2 was purchased from Cowellmedi (Seoul, Korea). hPDL cells were purchased from ScienCell™ Research Laboratories (Carlsbad, CA, USA) and maintained in alpha-minimum essential medium (αMEM) supplemented with 10% FBS and antibiotics. Osteogenic differentiation of hPDL cells was induced by osteogenic supplements (50 μg/mL ascorbic acid, 10 mM β-glycerophosphate and 0.1 μM dexamethasone).

Glucose concentration in DMEM and αMEM was 5 mM. To induce excessive *O*-GlcNAcylation, C2C12 and hPDL cells were exposed to the indicated glucose (10–60 mM), glucosamine (1–5 mM), or *N*-acetylglucosamine (1–5 mM) concentrations. Expression plasmids for human OGT and OGA were kindly provided by Prof. Jin Won Cho from Yonsei University and were transiently transfected into C2C12 cells using Lipofectamine 2000 reagent (Invitrogen, Waltham, MA, USA) [34].

### 4.2. Quantitative Real-Time Polymerase Chain Reaction (qRT-PCR) Analysis

Total RNA extraction and qRT-PCR was performed as previously described [35]. Target genes were normalized to the reference housekeeping gene glyceraldehyde 3-phosphate dehydrogenase (GAPDH). The mouse PCR primer sequences used for qRT-PCR were as follows: *Alpl* (ALP), (f) 5′-CCA ACT CTT TTG TGC CAG-3′ and (r) 5′-GGC TAC ATT GGT GTT GAG CTT TT-3′; *Runx2*, (f) 5′-TTC TCC AAC CCA CGA ATG CAC-3′ and (r) 5′-CAG GTA CGT GTG GTA GTG AGT-3′; *Sp7* (Osterix), (f) 5′-CCC ACC CTT CCC TCA CTC-3′ and (r) 5′-CCT TGT ACC ACG AGC CAT-3′; *Bglap2* (Osteocalcin), (f) 5′-CTG ACA AAG CCT TCA TGT CCA A-3′ and (r) 5′-GCG CCG GAG TCT GTT CAC TA-3′; *Col1a1* (f) 5′-GCT CCT CTT AGG GGC CAC T-3′ and (r) 5′-CCA CGT CTC ACC ATT GGG G-3′; *Ogt*, (f) 5′-CTG TCA CCC TTG ACC CAA AT-3′ and (r) 5′-ACG AAG ATA AGC TGC CAC AG-3′; *Mgea5*, (f) 5′-TGG AAG ACC TTG GGT TAT GG-3′ and (r) 5′-TGC TCA GCT TCT TCC ACT GA-3′; and *Gapdh*, (f) 5′-TCA ATG ACA ACT TTG TCA AGC-3′ and (r) 5′-CCA GGG TTT CTT ACT CCT TGG-3′. The human PCR primer sequences used for qRT-PCR were as follows: *ALPL*, (f) 5′-AAC TTC CAG ACC GGC TTG A-3′ and (r) 5′-TTG CCG CGT GTC TT-3′; *RUNX2*, (f) 5′-CAG ATG GGA CTG TGG CTG T-3′ and (r) 5′-GTG AAG ACG GTT ATG AAG G-3′; *SP7*, (f) 5′-ACC TAC CCA TCT GAC TTT GCT-3′ and (r) 5′-CCA CTA TTT CCC ACT GCC TTG-3′; *DLX5* (f) 5′-CAA CTT TGC CCG AGT CTT C-3′ and (r) 5′-GTT GAG AGC TTT GCC ATA GG-3′; and *GAPDH*, (f) 5′-CCA TCT TCC AGG AGC GAG ATC-3′ and (r) 5′-GCC TTC TCC ATG GTG GTG AA-3′.

### 4.3. Western Blot Analysis and Immunoprecipitation

Cell lysates for Western blot analysis were prepared using PRO-PREP^TM^ (iNtRON Biotechnology, Sungnam, Korea) and were briefly sonicated. To examine cellular global protein *O*-GlcNAcylation patterns, samples containing equal amounts of protein were subjected to sodium dodecyl sulfate (SDS)-polyacrylamide gel electrophoresis (SDS-PAGE) and transferred onto a polyvinylidene difluoride membrane. The membrane was blocked with 5% skim milk in Tris-buffered saline containing 0.1% Tween 20. The membrane was serially incubated with *O*-GlcNAc antibody, HRP-conjugated secondary antibody, and Sensi-view^TM^ Pico ECL Reagent (Lugen Sci. Inc., Buncheon, Korea), followed by the detection of chemiluminescence using a MicroChemi system (DNR; Jerusalem, Israel).

For immunoprecipitation, cell lysates were prepared using buffer containing 20 mM Tris-Cl (pH 7.5), 150 mM NaCl, 1 mM ethylenediaminetetraacetic acid(pH 8.0), 2% Brij-35, 2.5 mM sodium pyrophosphate, 1 mM β-glycerophosphate, 1 mM sodium orthovanadate, 1 mM phenylmethylsulfonyl fluoride, 1-µg/mL aprotinin, 1 µM leupeptin, and 1 µM pepstatin. Each 1 mg of protein sample was mixed with the Runx2 antibody and protein G agarose beads. The washed bead pellet was denatured by boiling in 2× SDS sample buffer, followed by SDS-PAGE and immunoblot analysis with *O*-GlcNAc and Myc antibodies.

Antibodies used for immunoprecipitation and Western blot analyses were as follows: *O*-GlcNAc antibody purchased from BioLegend (San Diego, CA, USA); Myc, Runx2, and actin antibodies purchased from Santa Cruz Biotechnology (Dallas, TX, USA); and HRP-conjugated secondary antibodies purchased from Thermo Fisher Scientific (Waltham, MA, USA).

### 4.4. ALP Staining, ALP Activity Assays, and Alizarin Red S Staining

At the end of the culture period, ALP staining was performed by employing an ALP staining kit (Sigma-Aldrich, St. Louis, MO, USA) according to the manufacturer’s instructions. ALP activity was assessed using a QuantiChrom^TM^ ALP assay kit (BioAssay Systems, Hayward, CA, USA) according to the manufacturer’s instructions. ALP activity was normalized to the total protein amount in the samples.

Matrix mineralization was examined by Alizarin red S staining. hPDL cells were incubated in the osteogenic medium for 21 days in the presence or absence of *N*-acetylglucosamine (2.5 mM). At the end of the culture period, the cells were fixed with 70% ethanol and stained with 2% Alizarin red S solution for 10 min at room temperature. After thorough rinsing with distilled water, the calcium deposition was quantified by the elution of the stain with 0.5 N HCl containing 5% SDS and the measurement of the optical density at 415 nm.

### 4.5. Luciferase Reporter Assays

C2C12 cells were seeded in a 96-well plate at a density of 1 × 10^4^ cells/well and, after overnight culture, the cells were transiently transfected with Myc-Runx2 expression plasmids and a Runx2 reporter plasmid (OSE-luc) using LipofectAMINE 2000 (Invitrogen, Carlsbad, CA, USA). In each transfection, 0.5 μL of LipofectAMINE 2000, 0.2 μg each of indicated expression plasmids and reporter plasmids, and 0.07 μg of Renilla luciferase plasmids were used. Luciferase activity was measured after an additional incubation for 24 h in the presence or absence of *O*-GlcNAcylation inducers and OGT inhibitor [36]. When indicated, OGT or OGA expression plasmids were co-transfected with Myc-Runx2 expression plasmids. Luciferase assays were performed using the Dual-Glo Luciferase Assay System (Promega, Madison, WI, USA) and the GloMax-Multi Detection System (Promega). The relative luciferase activity was calculated by dividing firefly luciferase activity by Renilla luciferase activity to normalize the transfection efficiency.

### 4.6. Statistical Analysis

All quantitative data are presented as means ± standard deviation. Statistical significance was analyzed by ANOVA with Tukey’s multiple comparison tests using Prism 6 (GraphPad Software Inc., La Jolla, CA, USA). A *p* value < 0.05 was considered statistically significant.
